# Vaginal Ecosystem During Gestation and Puerperium: Microbiota, Dysbiosis, Infection and Fetal–Maternal Implications

**DOI:** 10.3390/microorganisms14091930

**Published:** 2026-09-01

**Authors:** Antonio Braga, Gustavo Ribeiro Lima, Fernanda da Costa Negraes, Maria Vitória Moura Fajardo, Karine Mello Duvivier, Luis Fernando Lima Bueno, Susana Cristina Aidé Viviani Fialho, Edward Araujo Júnior, Jorge Rezende-Filho

**Affiliations:** 1 Department of Gynecology and Obstetrics, School of Medicine, Maternity School, Federal University of Rio de Janeiro, Rio de Janeiro 22240-003, RJ, Brazil; bragamed@yahoo.com.br (A.B.); rezendef@me.ufrj.br (J.R.-F.); 2 Department of General and Specialized Surgery, School of Medicine and Surgery, Federal University of the State of Rio de Janeiro, Rio de Janeiro 20270-004, RJ, Brazil; 3 Postgraduate Program in Applied Health Sciences, University of Vassouras, Vassouras 27700-000, RJ, Brazil; 4 Service of Gynecology and Obstetrics, Antônio Pedro University Hospital, Fluminense Federal University, Niterói 24070-090, RJ, Brazil; gustavoribeirolima@hotmail.com (G.R.L.); mariavitoriafajardo06@gmail.com (M.V.M.F.); karineduvivier@id.uff.br (K.M.D.); susanaaide@id.uff.br (S.C.A.V.F.); 5 Discipline of Gynecology and Obstetrics, Santo Amaro University (UNISA), São Paulo 04829-300, SP, Brazil; luisfernandolimabuenooficial@gmail.com; 6 Department of Maternal and Child Health, School of Medicine, Fluminense Federal University, Niterói 24070-090, RJ, Brazil; 7 Department of Obstetrics, Paulista School of Medicine, Federal University of São Paulo, São Paulo 04023-062, SP, Brazil; 8 Discipline of Woman Health, Municipal University of São Caetano do Sul (USCS), São Caetano do Sul 09521-160, SP, Brazil

**Keywords:** pregnancy, vaginal microbiome, vaginal dysbiosis, bacterial vaginosis, aerobic vaginitis, precision medicine

## Abstract

The vaginal ecosystem undergoes profound physiological adaptations during pregnancy and the puerperium through dynamic interactions among the vaginal microbiota, epithelial barrier, local immune system, and hormonal milieu. Disruption of this homeostasis may lead to vaginal dysbiosis and infection, with potential consequences for maternal, fetal, and neonatal health. This narrative review aimed to provide a comprehensive and updated overview of the vaginal ecosystem throughout pregnancy and the puerperium, integrating current evidence on physiological changes in the vaginal microbiota, mechanisms of dysbiosis, major vaginal infections, diagnostic approaches, therapeutic management, and maternal–fetal implications. A structured literature search was conducted in PubMed/MEDLINE and Scopus through July 2026. Priority was given to recent systematic reviews, meta-analyses, international clinical guidelines, randomized clinical trials, and observational studies addressing the vaginal microbiota, dysbiosis, bacterial vaginosis, vulvovaginal candidiasis, trichomoniasis, aerobic vaginitis, pregnancy, and the puerperium. Pregnancy is generally characterized by a stable, low-diversity, *Lactobacillus*-dominated vaginal microbiota, whereas the postpartum period is associated with reduced *Lactobacillus* abundance, increased microbial diversity, and gradual restoration of eubiosis. Disruption of this ecosystem promotes biofilm formation, microbial persistence, inflammation, and ascending infection. Bacterial vaginosis, vulvovaginal candidiasis, trichomoniasis, and aerobic vaginitis represent the major vaginal infections during pregnancy and the puerperium and have varying associations with adverse outcomes, including preterm birth, preterm premature rupture of membranes, chorioamnionitis, postpartum infection, and neonatal morbidity. Molecular diagnostics and microbiome profiling have improved etiological characterization, while emerging microbiome-directed interventions offer potential strategies for restoring vaginal homeostasis. Current evidence supports an increasingly ecosystem-centered approach to vaginal health during pregnancy and the puerperium. Accurate etiological diagnosis and evidence-based treatment remain essential, while preservation and restoration of vaginal homeostasis may represent important complementary objectives for improving maternal, fetal, and neonatal outcomes.

## 1. Introduction

The vaginal ecosystem is a dynamic biological environment in which the resident microbiota interacts continuously with the vaginal epithelium, local immune system, hormonal milieu, and host-related factors to maintain genital tract homeostasis. In healthy reproductive-age women, this ecosystem is commonly characterized by a relatively low-diversity microbiota dominated by *Lactobacillus* species, which contribute to the maintenance of an acidic vaginal environment, epithelial barrier integrity, and resistance to colonization by potentially pathogenic microorganisms [1,2,3,4,5,6]. The composition and stability of this ecosystem, however, are influenced by several biological and environmental factors, including hormonal status, reproductive stage, sexual and behavioral factors, antimicrobial exposure, ethnicity, and geographic background [1,2,3,4,5,6].

Disruption of this ecological equilibrium, generally referred to as vaginal dysbiosis, involves not only changes in microbial composition but also functional alterations in the vaginal microenvironment. Depletion of protective *Lactobacillus* species and expansion of anaerobic or facultative microorganisms may be accompanied by increased vaginal pH, biofilm formation, epithelial barrier dysfunction, and local inflammatory activation, thereby favoring microbial persistence and vaginal infection [1,2,3,4,5,6]. Clinically, these disorders are among the most frequent causes of gynecological consultation, commonly presenting with vaginal discharge, malodor, pruritus, burning, and genital discomfort. Their clinical relevance extends beyond local symptoms, particularly during pregnancy, when disruption of vaginal homeostasis has been associated with adverse reproductive and obstetric outcomes [1,2,3,4,5,6,7,8,9,10].

Pregnancy and the puerperium are characterized by profound physiological adaptations that directly influence the vaginal ecosystem. Hormonal fluctuations, immune modulation, and changes in the composition and metabolic activity of the vaginal microbiota contribute to maintaining maternal–fetal homeostasis but may also predispose to vaginal dysbiosis and infection. Among the conditions associated with disruption of vaginal homeostasis, bacterial vaginosis, vulvovaginal candidiasis, trichomoniasis, and aerobic vaginitis are the most clinically relevant. These disorders have been associated with adverse maternal, fetal, and neonatal outcomes, including preterm birth, premature rupture of membranes, chorioamnionitis, postpartum infection, and neonatal morbidity, highlighting their particular importance in obstetric care [1,2,3,4,5,6,7,8,9,10,11]. Despite their high prevalence and clinical relevance, the etiological diagnosis of vaginal infections remains challenging because of the considerable overlap in clinical manifestations, the frequent occurrence of mixed infections, the existence of noninfectious causes of vaginal symptoms, and the marked interindividual variability of the vaginal microbiota [3,6,7,8].

During pregnancy, accurate diagnosis is particularly important because early recognition and appropriate management may reduce the risk of obstetric complications in selected women. Although routine screening for bacterial vaginosis in asymptomatic pregnant women, including those at high risk of preterm delivery, is not recommended by the Centers for Disease Control and Prevention, symptomatic women should be evaluated and treated [2]. Moreover, increasing bacterial and antifungal resistance, together with the expanding role of molecular diagnostic methods, has transformed the contemporary approach to vaginal infections, emphasizing the need for precise etiological diagnosis and evidence-based management [3,4].

Although several reviews have addressed the vaginal microbiome in pregnancy, the available literature remains largely fragmented, with most publications focusing on specific aspects such as microbiome composition, individual vaginal infections, preterm birth, or particular diagnostic and therapeutic approaches. Moreover, the postpartum period and its distinctive ecological transition remain comparatively less integrated into reviews of the pregnancy-associated vaginal ecosystem. A comprehensive clinical framework connecting physiological microbiome adaptations, mechanisms of dysbiosis, the major vaginal infections, maternal–fetal and neonatal consequences, contemporary diagnostic strategies, evidence-based treatment, and emerging microbiome-directed interventions across the pregnancy–postpartum continuum is still lacking. This fragmentation may limit the translation of rapidly expanding microbiome research into practical obstetric care. The present review was therefore designed to bridge this gap by integrating these dimensions within a single ecosystem-centered and clinically oriented framework.

This narrative review provides a comprehensive and updated overview of the vaginal ecosystem throughout gestation and the postpartum period, integrating current knowledge on vaginal microbiota, mechanisms of dysbiosis, the principal vaginal infections, diagnostic strategies, evidence-based management, and maternal–fetal implications. By discussing these components within a unified framework, we aim to provide clinicians with a practical, evidence-based overview of the physiological and pathological changes occurring throughout the pregnancy–puerperium continuum and their implications for maternal and neonatal health.

## 2. Methods

As this study was designed as a narrative review with a structured literature search, no formal assessment of risk of bias or certainty of evidence was performed, and the study did not follow PRISMA reporting guidelines. This methodological approach was selected because the objective was to provide a broad, clinically oriented synthesis of the vaginal ecosystem across the pregnancy–postpartum continuum rather than to address a narrowly defined research question through a systematic review or meta-analysis. As no human participants, individual patient data, or animal subjects were involved, institutional ethics committee approval and informed consent were not required. A structured literature search was performed in PubMed/MEDLINE and Scopus through July 2026 using combinations of Medical Subject Headings (MeSH) and free-text terms. The search strategy combined terms related to the vaginal ecosystem (“*vaginal microbiota*” OR “*vaginal microbiome*” OR “*vaginal ecosystem*” OR dysbiosis) with terms related to the principal vaginal infections (“*bacterial vaginosis*” OR “*vulvovaginal candidiasis*” OR trichomoniasis OR “*aerobic vaginitis*”) and pregnancy-related conditions (*pregnancy* OR *pregnant* OR *gestation* OR *postpartum* OR *puerperium*), using the Boolean operators AND and OR. Additional searches included terms related to clinical outcomes and management, including *diagnosis*, *treatment*, *molecular diagnostics*, and *preterm birth*. Reference lists of relevant publications were also manually screened to identify additional eligible studies.

Priority was given to recent systematic reviews, meta-analyses, international clinical guidelines, randomized clinical trials, and observational studies. Narrative reviews were considered when they provided comprehensive background or contextual information relevant to the topics addressed. Classic landmark studies were also included when necessary to provide historical context or to describe concepts that remain fundamental to the understanding of vaginal microbiota and dysbiosis. Evidence was selected according to its methodological quality, clinical relevance, and consistency with recommendations from major international scientific societies.

The retrieved evidence was synthesized narratively to integrate current knowledge on physiological adaptations of the vaginal ecosystem in pregnant and postpartum women, mechanisms of dysbiosis, clinical manifestations, diagnostic approaches, therapeutic strategies, and maternal–fetal outcomes.

## 3. Vaginal Ecosystem and Its Physiological Adaptations

The vaginal ecosystem is a dynamic biological system shaped by continuous interactions between microbiota, the vaginal epithelium, metabolism, sexual hormones, immunity, mucosa, and environmental factors, which act together to maintain homeostasis in the female genital tract. Contrary to the traditional view that the vaginal microbiota is exclusively composed of microorganisms, it has been suggested that its composition is the result of the relationship between the host and the microbiota and therefore that its stability depends on epithelial integrity, metabolic activity, and the immune response [12,13,14].

During menarche in healthy women, the microbiota is predominantly composed of microorganisms from the *Lactobacillus* species such as *Lactobacillus crispatus*, *L. jensenii*, *L. gasseri* and *L. iners*. The abundance of these microorganisms varies according to genetic, hormonal, and environmental factors. These bacteria play an important role in the maintenance of vaginal health by producing lactic acid, hydrogen peroxide, bacteriocins, and other bioactive compounds capable of inhibiting pathogen adhesion, modulating local inflammation, and strengthening the epithelial barrier [13,14,15,16].

As a result, the physiological pH of the vaginal environment remains between 3.8 and 4.5, which is essential to limit the growth of potential pathogenic microorganisms and to preserve the ecological balance. The glycogen present on the epithelial cells is the main metabolic substrate of the lactobacilli, providing continuous production of metabolites that protect and favor the maintenance of the microbiota balance, a defense mechanism against gynecological and obstetric infections [14,15,16].

**Figure 1:**
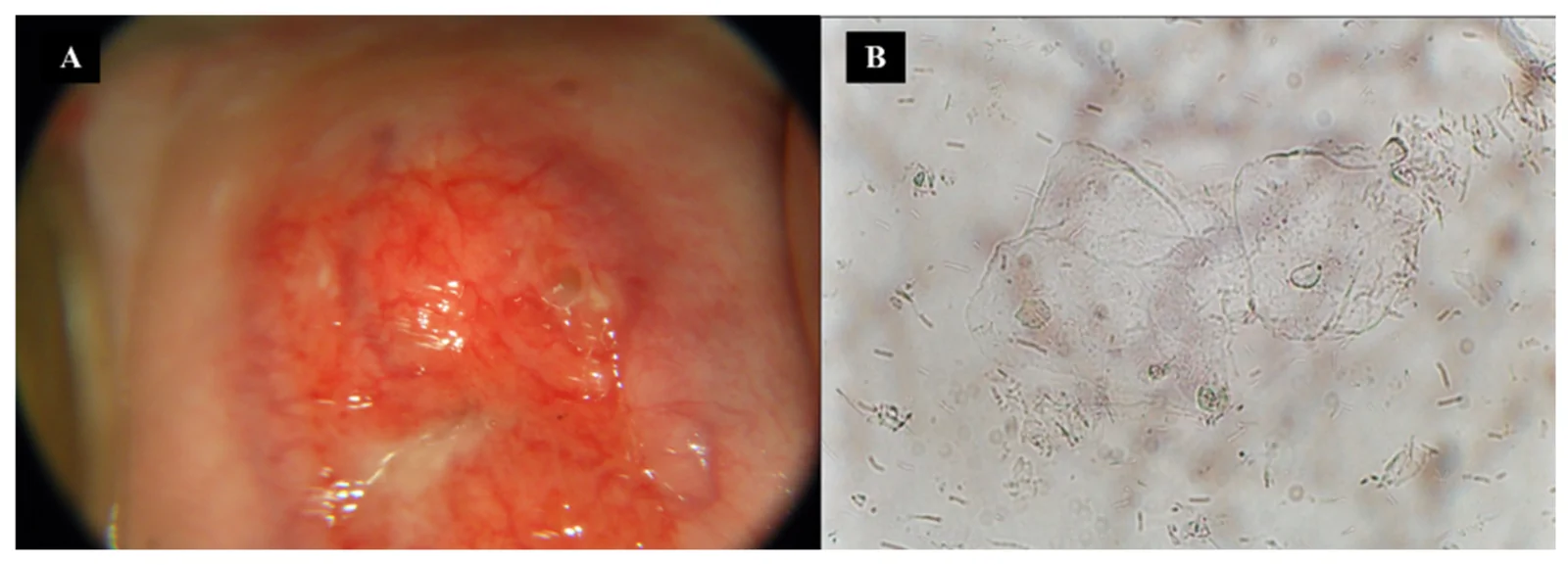
Representative clinical and microscopic features of the physiological vaginal ecosystem (vaginal eubiosis). (**A**) Speculum examination demonstrating physiological translucent/milky vaginal secretions, mild peri-orificial cervical hyperemia, and Nabothian cysts. Vaginal pH was within the normal physiological acidic range (3.8–4.5). (**B**) Wet mount microscopy (×400) showing predominantly superficial and intermediate epithelial cells with abundant *Lactobacillus* spp., consistent with a Lactobacillus-dominated vaginal microbiota and preserved vaginal homeostasis. Clinical images were obtained from patients under the care of the authors and are presented with informed consent for publication.

The maintenance of vaginal homeostasis can also be observed through routine clinical evaluation. In healthy reproductive-age women, speculum examination typically reveals physiological vaginal secretions without signs of significant inflammation, while bedside assessment demonstrates an acidic vaginal pH (3.8–4.5) [1,10]. Wet mount microscopy complements the clinical examination by confirming the predominance of superficial and intermediate epithelial cells together with abundant *Lactobacillus* spp., findings consistent with a eubiotic vaginal ecosystem [10,14]. These clinical and microscopic features provide the biological basis for understanding how disruption of vaginal homeostasis progresses to dysbiosis and vaginal infection [14]. The characteristic features of vaginal eubiosis are illustrated in Figure 1.

The development of next-generation sequencing technologies has allowed the characterization of vaginal microbiome into Community State Types (CSTs), a classification based on the predominant bacterial species [12]. CSTs I, II, III, and V are predominantly composed of *Lactobacillus crispatus*, *L. gasseri*, *L. iners*, and *L. jensenii*, respectively, whereas CST IV is characterized by a marked reduction in lactobacilli and the predominance of anaerobic bacteria, including *Gardnerella*, *Prevotella*, *Atopobium*, and other species associated with dysbiosis [12,13,14]. Lactobacillus-dominated microbiota is associated with lower bacterial diversity, whereas highly diverse anaerobic communities are associated with disruption of vaginal homeostasis [14] and increased susceptibility to gynecological and obstetric infections [13,14,15].

Among the *Lactobacillus*-dominated community states, the distinction between *L. crispatus*- and *L. iners*-dominated communities is particularly relevant. CST I, dominated by *L. crispatus*, is generally associated with greater ecological stability, sustained lactic acid production, lower vaginal pH, and reduced inflammatory activity. In contrast, CST III, dominated by *L. iners*, may occur in apparently healthy women but is considered less stable and more prone to transition toward dysbiosis. *Lactobacillus*-depleted, highly diverse communities, particularly CST IV, are characterized by enrichment of anaerobic taxa and are more frequently associated with bacterial vaginosis, vaginal inflammation, and adverse reproductive and obstetric outcomes [13,14,15]. Thus, *Lactobacillus* dominance should not be considered biologically uniform, as the predominant species and overall community structure may substantially influence vaginal ecosystem stability and clinical risk.

Importantly, the composition and diversity of the vaginal microbiota vary substantially across the female lifespan and should be interpreted according to the hormonal and reproductive context. Rather than being defined by a fixed number of bacterial species, the vaginal ecosystem is characterized by marked differences in bacterial richness, relative abundance, and community structure. In reproductive-age women, *Lactobacillus* spp. frequently account for the majority of the vaginal bacterial community.

Pregnancy further modifies this ecological pattern. Compared with the nonpregnant state, the vaginal microbiota during pregnancy generally exhibits reduced richness and diversity, increased *Lactobacillus* dominance, and greater temporal stability [6,14]. These changes become particularly evident as pregnancy progresses and are considered part of the physiological adaptation of the lower genital tract to gestation. *L. crispatus*-dominated communities are generally associated with the greatest stability [14], whereas *L. iners*-dominated and Lactobacillus-depleted communities tend to show greater ecological instability and a higher probability of transition toward dysbiosis [14,15].

The transition after delivery is particularly pronounced [6,14]. Within the postpartum period, the abrupt decline in estrogen levels and the associated reduction in vaginal epithelial glycogen are accompanied by a decrease in *Lactobacillus* abundance and an increase in bacterial richness and diversity [6,14]. Consequently, communities enriched in anaerobic bacteria become substantially more frequent than during pregnancy [6]. This postpartum microbial configuration may persist for several weeks or months, particularly during breastfeeding, when lactational hypoestrogenism delays restoration of the Lactobacillus-dominated ecosystem [14,15].

Recovery of the vaginal microbiome after delivery is therefore a gradual rather than immediate process. The early postpartum period is typically characterized by reduced *Lactobacillus* dominance and expansion of more diverse anaerobic communities, and restoration toward a pregnancy-like, *Lactobacillus*-dominated state may require several months. The timing of this recovery varies considerably among women and is strongly influenced by the postpartum hormonal environment. In breastfeeding women, hyperprolactinemia suppresses ovarian estrogen production, resulting in persistent hypoestrogenism, reduced vaginal epithelial glycogen availability, higher vaginal pH, and delayed recolonization by *Lactobacillus* species. Consequently, *Lactobacillus* depletion and increased microbial diversity may persist throughout lactation, whereas recovery may occur earlier after resumption of ovarian activity and increasing estrogen exposure. These observations indicate that postpartum microbiome recovery should be interpreted as a dynamic, hormonally influenced process rather than as a defined transition occurring at a uniform time after delivery [17,18,19,20,21,22].

In microbiome studies, relative abundance refers to the proportion of sequencing reads assigned to a given bacterial taxon relative to the total bacterial reads detected in a sample. Quantitatively, longitudinal data illustrate the magnitude of these changes across the pregnancy–postpartum transition. *Lactobacillus*-dominated communities, defined as those in which *Lactobacillus* represented >50% of the bacterial community, were observed in approximately 79% and 78% of women during the first and third trimesters, respectively, whereas approximately 77% of postpartum communities exhibited low proportions of *Lactobacillus*. At the species level, the mean relative abundance of *L. crispatus* decreased from 49.1% and 44.0% during the first and third trimesters, respectively, to 6.7% postpartum, while *L. jensenii* decreased from 16.2–17.4% to 0% and *L. gasseri* from 10.2–13.9% to 0.6% [17]. These quantitative changes illustrate the marked loss of *Lactobacillus* predominance that characterizes postpartum remodeling of the vaginal microbiota.

Therefore, quantitative comparisons of the vaginal microbiota across life stages should preferentially be expressed in terms of relative abundance, bacterial diversity, and prevalence of specific community structures rather than as an absolute number of bacterial species. Reported estimates vary considerably according to ethnicity, geographic population, sequencing platform, sampling methodology, and the taxonomic resolution employed. These methodological and population-related differences should be considered when comparing microbiome studies and preclude the establishment of a single universal numerical definition of the normal vaginal microbiota.

To understand the physiological adaptations of the vaginal ecosystem during pregnancy and the puerperium, it is important to recognize that the vaginal microbiota undergoes dynamic changes throughout a woman’s life, largely driven by fluctuations in sex hormone concentrations. During childhood and the prepubertal period, low estrogen levels result in limited epithelial glycogen deposition, a higher vaginal pH, and a diverse microbiota with low *Lactobacillus* abundance. With the onset of puberty and ovarian function, increasing estrogen concentrations promote glycogen accumulation, favoring the establishment of a *Lactobacillus*-dominant microbiota, a more acidic vaginal environment, and greater microbial stability. During the menstrual cycle, modest fluctuations in microbial composition occur, particularly during menstruation, when transient increases in vaginal pH are associated with a temporary rise in bacterial diversity that rapidly returns to baseline during subsequent phases of the cycle. Conversely, menopause is characterized by estrogen deficiency, reduced glycogen availability, loss of *Lactobacillus* predominance, and increased microbial diversity [16,17,23,24]. Collectively, these physiological changes establish the biological framework through which alterations in the vaginal ecosystem may progress to dysbiosis, vaginal infection, and subsequent maternal–fetal complications during pregnancy and the puerperium.

Pregnancy is characterized by extensive hormonal, immunological, and metabolic adaptations that reshape the vaginal ecosystem into a highly stable, *Lactobacillus*-dominated environment. Rising estrogen and progesterone concentrations stimulate glycogen accumulation within the vaginal epithelium, providing an abundant substrate for *Lactobacillus* metabolism. Consequently, the vaginal microbiota becomes less diverse and increasingly enriched with protective species, particularly *Lactobacillus crispatus* and *L. gasseri*, while anaerobic bacteria associated with dysbiosis are suppressed. These changes maintain an acidic vaginal pH and reinforce the natural defense mechanisms of the lower genital tract. Beyond taxonomic shifts, pregnancy also induces functional remodeling of the vaginal microbiome, characterized by enhanced lactate production and reduced concentrations of metabolites associated with bacterial vaginosis, thereby strengthening antimicrobial activity and contributing to the preservation of the fetal–maternal interface [17,18,19,23,24].

In parallel with these microbial adaptations, pregnancy induces finely regulated modulation of the mucosal immune system that balances maternal immune tolerance toward the semi-allogeneic fetus with effective protection against invading pathogens. The coordinated interaction among reproductive hormones, the vaginal microbiota, and the local immune response preserves epithelial barrier integrity, limits excessive inflammatory activation, and restricts pathogen colonization. Consequently, vaginal communities dominated by *Lactobacillus*, particularly *L. crispatus*, have consistently been associated with greater microbial stability, lower rates of dysbiosis, and more favorable obstetric outcomes [18,19,20,23].

**Figure 2:**
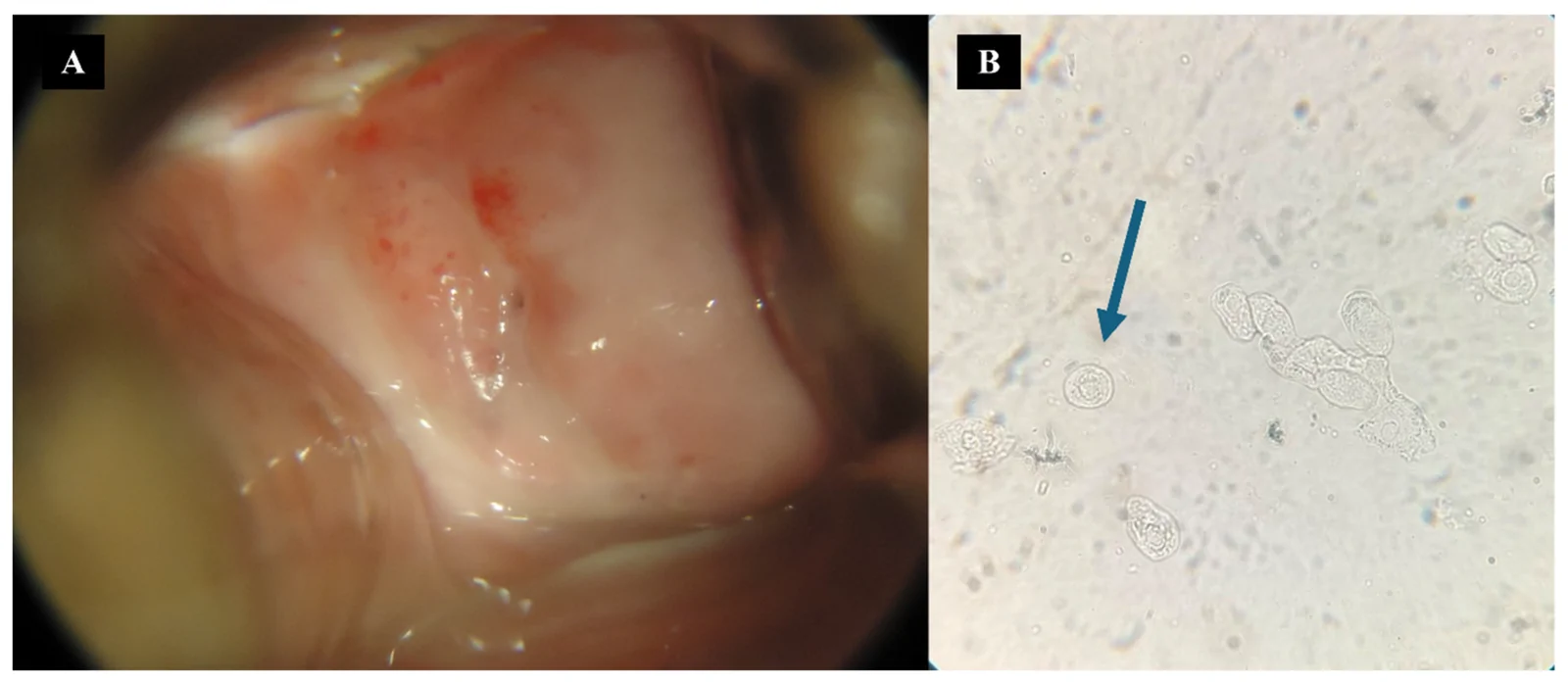
Representative clinical and microscopic findings in a breastfeeding postpartum woman presenting with dyspareunia secondary to hypoestrogenic vaginal atrophy. (**A**) Speculum examination demonstrating a pale, atrophic vaginal epithelium with petechial hemorrhages induced by speculum contact, consistent with increased epithelial fragility. Vaginal pH was >4.5, and the amine (whiff) test was negative. (**B**) Fresh saline wet mount microscopy (×400) showing a parabasal epithelial cell (blue arrow), a characteristic finding of hypoestrogenism associated with postpartum vaginal atrophy. The predominance of parabasal cells reflects impaired epithelial maturation resulting from estrogen deficiency and contributes to the increase in vaginal pH and depletion of *Lactobacillus* spp. Clinical images were obtained from patients under the care of the authors and are presented with informed consent for publication.

The postpartum period represents a rapid transition from this highly stable ecosystem to a more diverse microbial community. Following delivery, the abrupt decline in estrogen concentrations, further accentuated by lactation-associated hyperprolactinemia, markedly reduces epithelial glycogen availability, thereby limiting the metabolic substrate required for *Lactobacillus* proliferation [14,17]. As a result, vaginal pH increases, *Lactobacillus* abundance declines, and anaerobic bacterial diversity transiently expands [17]. These physiological postpartum changes are illustrated in Figure 2, which demonstrates the characteristic clinical and microscopic features of lactational hypoestrogenic vaginal atrophy.

Although these changes are physiological, longitudinal studies have shown that restoration of a eubiotic vaginal microbiota is gradual and may remain incomplete for several months after childbirth. Persistent hypoestrogenism during lactation also contributes to the development of the genitourinary syndrome of lactation, characterized by vaginal dryness, dyspareunia, reduced lubrication, and epithelial fragility, changes that may impair quality of life and compromise local defense mechanisms. Understanding these physiological adaptations provides the biological basis for recognizing how disruption of vaginal homeostasis predisposes to dysbiosis, vaginal infections, and adverse maternal and neonatal outcomes during pregnancy and the puerperium [18,19,20,21,22].

**Figure 3:**
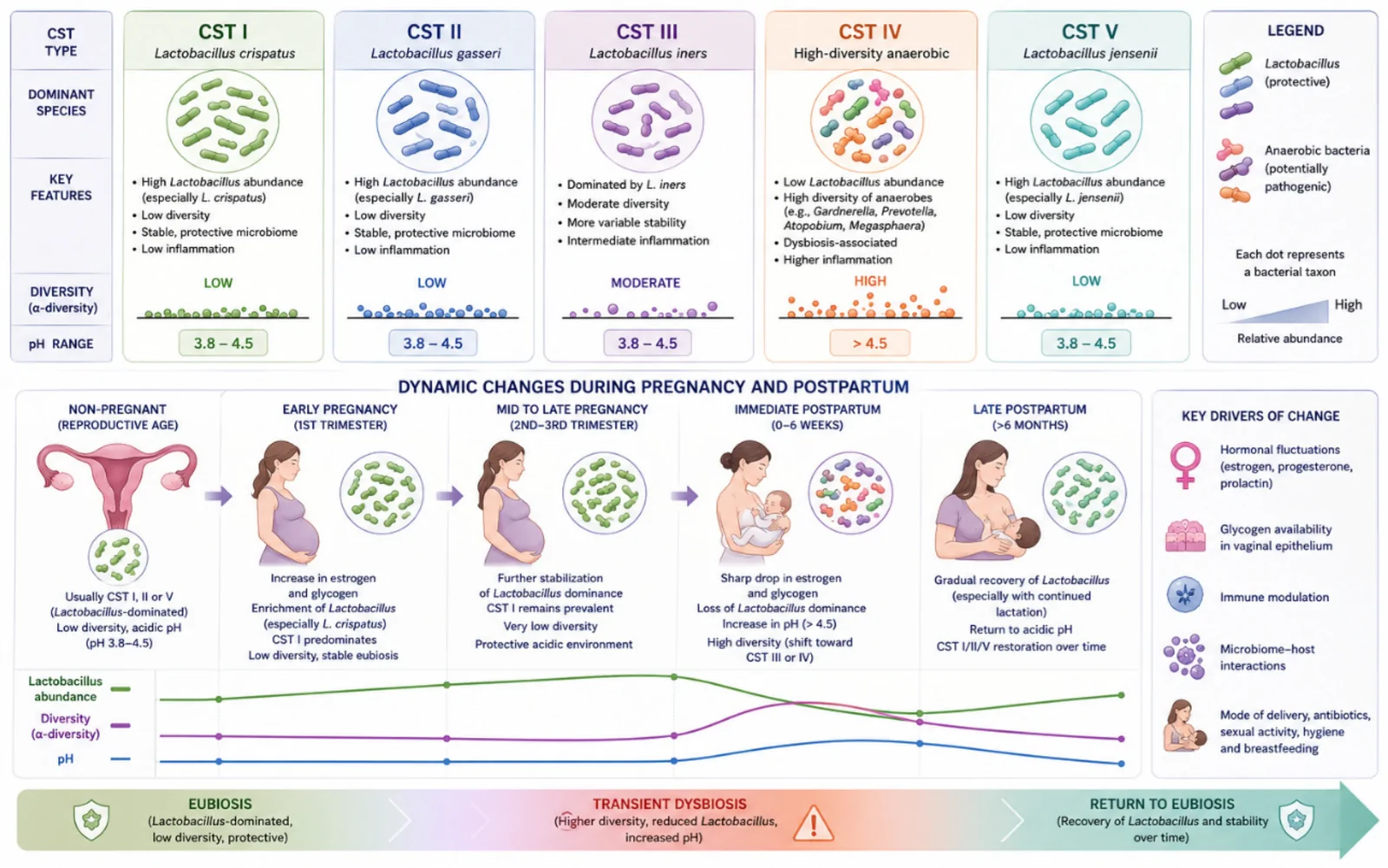
Physiological remodeling of the vaginal ecosystem across the female lifespan, with emphasis on pregnancy and the puerperium. Pregnancy is characterized by a stable *Lactobacillus*-dominated, low-diversity microbiota, whereas the postpartum period is associated with transient loss of *Lactobacillus* predominance, increased anaerobic diversity, elevated vaginal pH, and gradual restoration of eubiosis. Hormonal fluctuations, glycogen availability, and local immune modulation are the principal drivers of these adaptations [12,14,15,17,18,20,23].

The physiological evolution of the vaginal ecosystem across the female lifespan and the distinctive adaptations occurring during pregnancy and the puerperium are summarized in Figure 3. Based on the CST classification of the vaginal microbiome, the figure illustrates the dynamic interplay between hormonal fluctuations, microbial composition, immune modulation, and vaginal pH across different physiological stages.

## 4. From Vaginal Homeostasis to Dysbiosis During Pregnancy and the Puerperium

Vaginal dysbiosis represents a disruption of the physiological homeostasis that maintains the vaginal ecosystem in a stable, *Lactobacillus*-dominated state. Rather than a simple shift in microbial composition, dysbiosis encompasses quantitative, qualitative, and functional alterations in the vaginal microbiota, characterized by depletion of protective *Lactobacillus* species; expansion of anaerobic bacteria; reduced production of lactic acid, hydrogen peroxide, and bacteriocins; and a consequent increase in vaginal pH. Collectively, these changes weaken the intrinsic defense mechanisms of the vaginal mucosa, facilitating colonization by opportunistic microorganisms, persistence of local inflammation, ascending infection of the upper genital tract, and the development of adverse obstetric outcomes [25,26,27,28].

The dysbiotic vaginal microbiota is typically enriched with anaerobic organisms, including *Gardnerella vaginalis*, *Atopobium vaginae*, *Prevotella bivia*, *Prevotella timonensis*, *Megasphaera lornae*, *Megasphaera hutchinsoni*, *Sneathia amnii*, and *Sneathia sanguinegens*. However, dysbiosis extends beyond taxonomic changes and involves profound functional remodeling of the vaginal ecosystem. Loss of Lactobacillus dominance, particularly the depletion of protective species such as *Lactobacillus crispatus*, *Lactobacillus gasseri*, and *Lactobacillus jensenii*, reduces lactic acid production and other protective metabolic functions, creating a permissive microenvironment that favors microbial persistence, chronic inflammation, and increased susceptibility to infection. Because Lactobacillus abundance varies according to population and analytical method, no universally accepted quantitative threshold defines this depletion; rather, vaginal dysbiosis is characterized by a marked reduction in the relative abundance or dominance of Lactobacillus species, accompanied by increased microbial diversity and expansion of anaerobic taxa. Although pregnancy is generally characterized by remarkable microbial stability, disruption of this equilibrium has consistently been associated with increased maternal and neonatal morbidity [25,26,27,28].

Vaginal dysbiosis encompasses a broad spectrum of alterations in microbial composition, diversity, metabolic activity, and host–microbiota interactions and is not uniformly associated with biofilm formation. Polymicrobial biofilm formation is particularly characteristic of bacterial vaginosis, in which *Gardnerella vaginalis* plays a pivotal role by initiating epithelial adhesion and facilitating the sequential recruitment of other anaerobic microorganisms, ultimately generating a complex extracellular matrix firmly adherent to the vaginal epithelium. This biofilm architecture impairs antimicrobial penetration, protects microorganisms from host immune defenses, and contributes to the persistence and recurrence of bacterial vaginosis. Thus, although biofilm formation represents an important pathogenic mechanism in specific dysbiotic conditions, particularly bacterial vaginosis, it should not be regarded as a universal feature of vaginal dysbiosis [25,26,29]. The interaction between the vaginal microbiota and the mucosal immune system is fundamental for maintaining vaginal homeostasis and largely determines the host response to dysbiosis. While commensal species such as *Lactobacillus crispatus* preserve epithelial barrier integrity and promote an anti-inflammatory immune environment, dysbiosis-associated bacteria, including *Prevotella* spp. and *Megasphaera* spp., activate pattern-recognition receptors and dendritic cells, triggering the production of pro-inflammatory cytokines and chemokines that perpetuate local inflammation and epithelial dysfunction [30,31,32].

The development of vaginal dysbiosis during pregnancy and the puerperium is influenced by a complex interplay of behavioral, clinical, obstetric, hormonal, and microbial factors. Previous bacterial vaginosis, multiple sexual partners, vaginal douching, indiscriminate antibiotic use, smoking, and adverse socioeconomic conditions have all been associated with an increased risk of dysbiosis. Likewise, a history of preterm birth, spontaneous miscarriage, premature rupture of membranes, and cesarean delivery has been linked to persistent alterations in the vaginal microbiome. Following childbirth, physiological postpartum remodeling, compounded by lactation-associated hypoestrogenism, delays the re-establishment of a *Lactobacillus*-dominated microbiota, thereby prolonging the period of increased susceptibility to dysbiosis and infection. Together, these mechanisms illustrate that vaginal dysbiosis is a multifactorial disorder arising from the complex interaction between microbial communities, host immunity, endocrine regulation, and environmental factors, providing the pathophysiological basis for the major vaginal infections and their maternal–fetal consequences [27,31,32,33,34,35].

**Figure 4:**
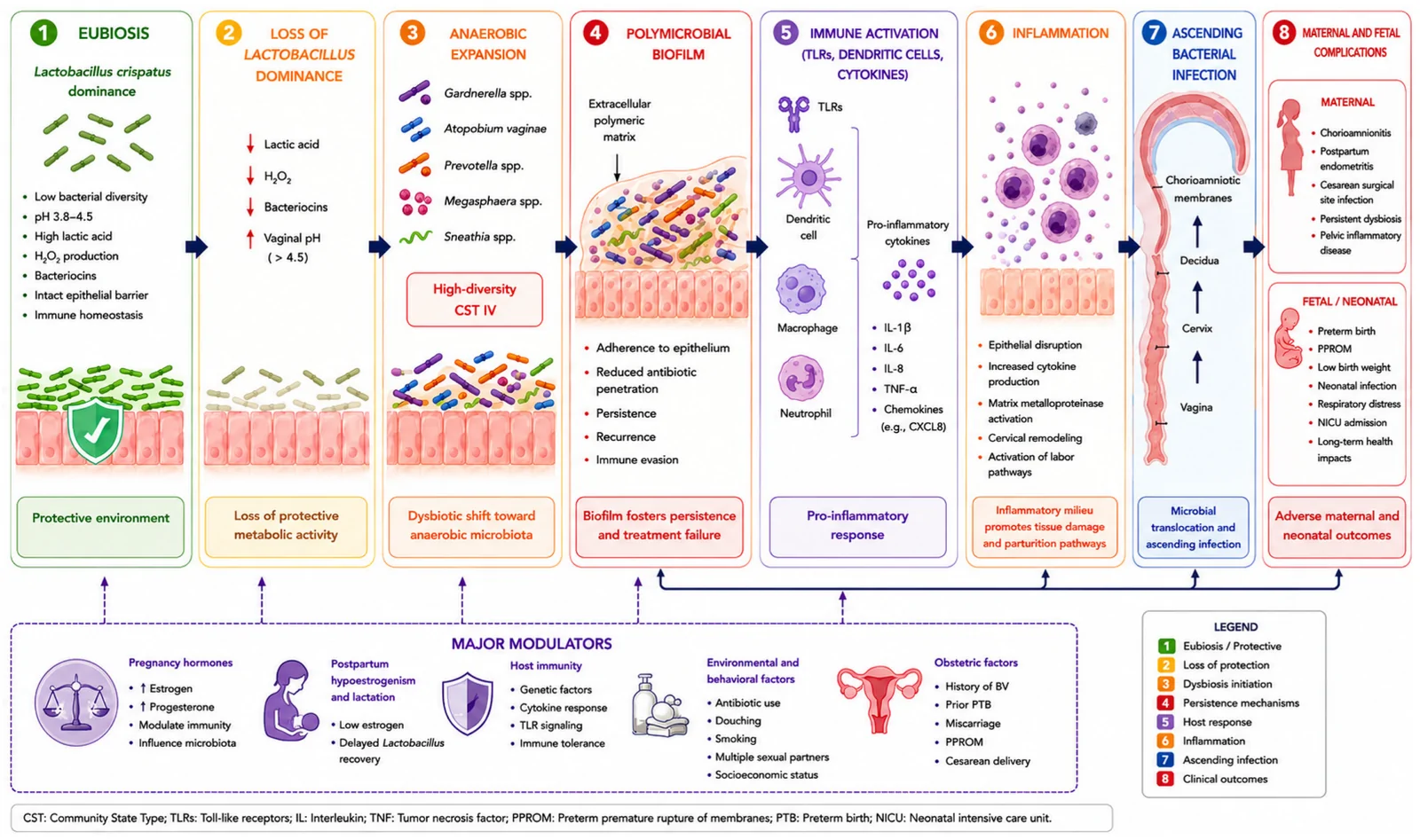
Pathophysiological Cascade of Vaginal Dysbiosis During Pregnancy and the Postpartum Period. Solid arrows indicate the sequential progression of the proposed pathophysiological pathway, whereas dotted arrows indicate the influence of major modulators on different stages of this process.

Figure 4 integrates the current understanding of the pathophysiological mechanisms underlying vaginal dysbiosis during pregnancy and the puerperium. The schematic model depicts the sequential transition from vaginal eubiosis to dysbiosis, emphasizing the interplay among microbial alterations, biofilm formation, host immune activation, epithelial barrier disruption, and the mechanisms leading to ascending infection and adverse maternal and neonatal outcomes [25,26,27,28,29,30,31,32,33,34,35]. The influence of pregnancy-associated endocrine adaptations and postpartum hypoestrogenism on each stage of this cascade is also illustrated.

## 5. Clinical Spectrum of Vaginal Infections During Pregnancy and the Puerperium

**Figure 5:**
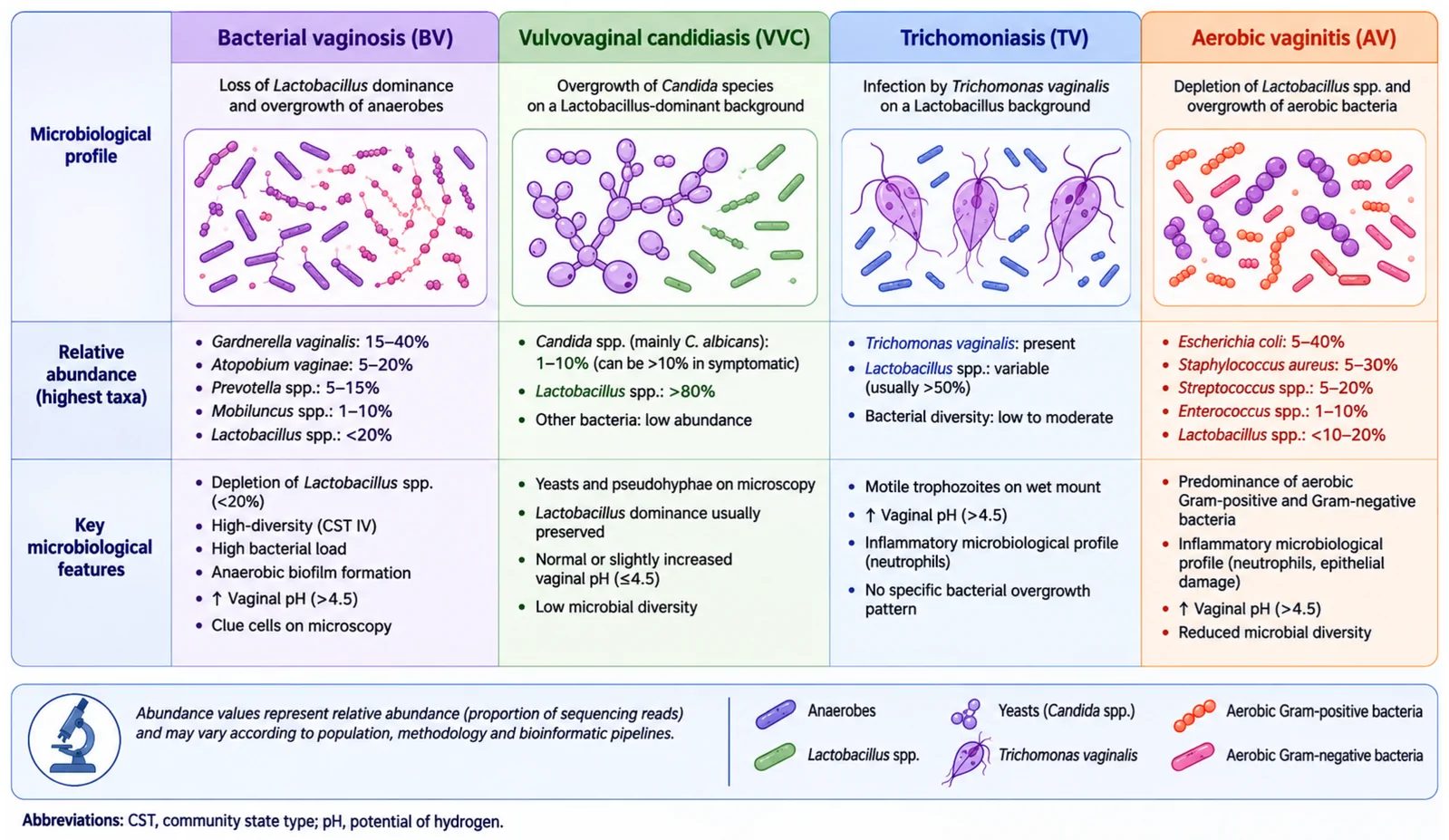
Comparative microbiological profiles of the major vaginal dysbiotic and infectious conditions. Schematic representation of the predominant microorganisms and characteristic alterations in the vaginal microbiota associated with bacterial vaginosis, vulvovaginal candidiasis, trichomoniasis, and aerobic vaginitis.

The major vaginal dysbiotic and infectious conditions addressed in this review are associated with distinct alterations in the vaginal microbial environment. Figure 5 provides a comparative graphical overview of the characteristic microbiological profiles of bacterial vaginosis, vulvovaginal candidiasis, trichomoniasis, and aerobic vaginitis, highlighting the predominant microorganisms, changes in Lactobacillus abundance, and principal microbiological features that distinguish these conditions.

### 5.1. Bacterial Vaginosis

Bacterial vaginosis (BV) is the most common cause of vaginal discharge among women of reproductive age and represents the principal clinical manifestation of vaginal dysbiosis. It is characterized by the replacement of a *Lactobacillus*-dominated vaginal microbiota by a polymicrobial anaerobic community enriched with organisms such as *Gardnerella* spp., *Fannyhessea vaginae*, *Prevotella* spp., *Sneathia* spp., and *Megasphaera* spp. This ecological shift is accompanied by increased vaginal pH, profound metabolic remodeling, and the formation of polymicrobial biofilms, all of which promote microbial persistence, resistance to treatment, and recurrent disease. The prevalence of BV varies considerably across populations according to demographic and behavioral characteristics, geographic setting, and the diagnostic criteria employed. Pregnancy itself is not consistently associated with a higher prevalence of BV compared with non-pregnant women of reproductive age [36,37,38,39].

**Figure 6:**
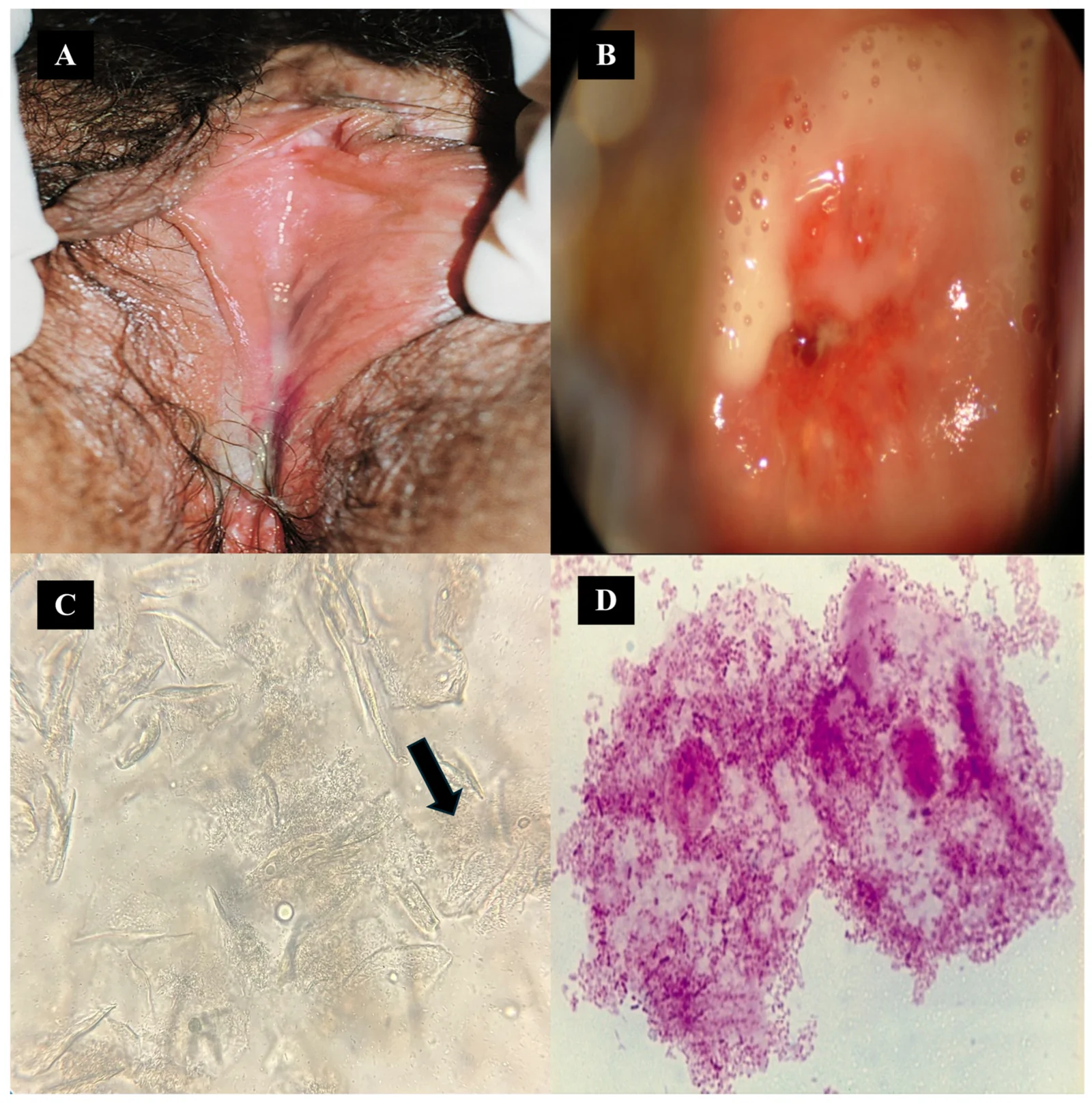
Representative clinical and laboratory findings in a pregnant woman presenting with malodorous vaginal discharge. (**A**) Clinical examination showing a normal vulva without erythema, edema, or other inflammatory changes. (**B**) Speculum examination demonstrating homogeneous grayish-white vaginal discharge with fine microbubbles. Vaginal pH was >4.5, and the amine (whiff) test was positive. (**C**) Wet mount microscopy (×400) showing numerous clue cells, depletion of *Lactobacillus* spp., and minimal inflammatory response, characteristic of bacterial vaginosis. The arrow indicates a representative clue cell, characterized by a squamous epithelial cell densely coated with adherent coccobacilli, which obscure its borders and give the cell a characteristic granular, stippled appearance. (**D**) Gram-stained vaginal smear showing a Nugent score of 9, characterized by depletion of *Lactobacillus* morphotypes and predominance of *Gardnerella/Bacteroides*-type bacteria, consistent with bacterial vaginosis. Clinical images were obtained from patients under the care of the authors and are presented with informed consent for publication.

Although many pregnant women remain asymptomatic, symptomatic BV typically presents with a thin, homogeneous grayish-white vaginal discharge accompanied by a characteristic fishy odor, which often becomes more noticeable after sexual intercourse or during menstruation. The diagnosis is traditionally established using the Amsel clinical criteria, which require at least three of four findings—homogeneous vaginal discharge; vaginal pH > 4.5; a positive amine (“whiff”) test, characterized by the release of a fishy odor after the addition of 10% potassium hydroxide (KOH) to vaginal secretions; and clue cells on microscopy—or the Gram stain-based Nugent scoring system, which assigns a score from 0 to 10 according to the relative abundance of *Lactobacillus*, *Gardnerella/Bacteroides*, and *Mobiluncus* morphotypes, with scores of 7–10 considered diagnostic of BV. Molecular diagnostic methods have also emerged as valuable complementary tools for characterizing the vaginal microbiota and identifying dysbiotic microbial profiles [36,37,38,39]. The polymicrobial biofilm characteristic of BV largely explains the high rates of persistence and recurrence observed despite appropriate antimicrobial therapy [36,37,38,39]. The integration of clinical examination, bedside microscopy, and Gram staining in the diagnosis of bacterial vaginosis is illustrated in Figure 6.

During pregnancy, BV has consistently been associated with an increased risk of adverse obstetric outcomes, including preterm birth, preterm premature rupture of membranes, chorioamnionitis, intra-amniotic infection, spontaneous abortion, low birth weight, postpartum endometritis, and neonatal intensive care unit admission. These complications are thought to result primarily from ascending microbial invasion and activation of inflammatory pathways at the maternal–fetal interface, underscoring the importance of early recognition and appropriate management, particularly in women with risk factors or suggestive clinical manifestations [27,28,36,40].

### 5.2. Vulvovaginal Candidiasis

Vulvovaginal candidiasis (VVC) is the second most common cause of vaginitis among women of reproductive age and results from the overgrowth of *Candida* species, predominantly *Candida albicans*, although infections caused by *Candida* species other than *C. albicans* have become increasingly recognized in recent years. Pregnancy is one of the strongest predisposing factors for VVC because elevated estrogen concentrations, increased glycogen deposition within the vaginal epithelium, and pregnancy-associated immune adaptations create a favorable environment for fungal proliferation and facilitate the transition of *Candida* from a commensal organism to an opportunistic pathogen. Unlike bacterial vaginosis, VVC typically develops in the presence of a physiologically acidic vaginal environment, with vaginal pH generally remaining within the normal range [41,42,43,44].

**Figure 7:**
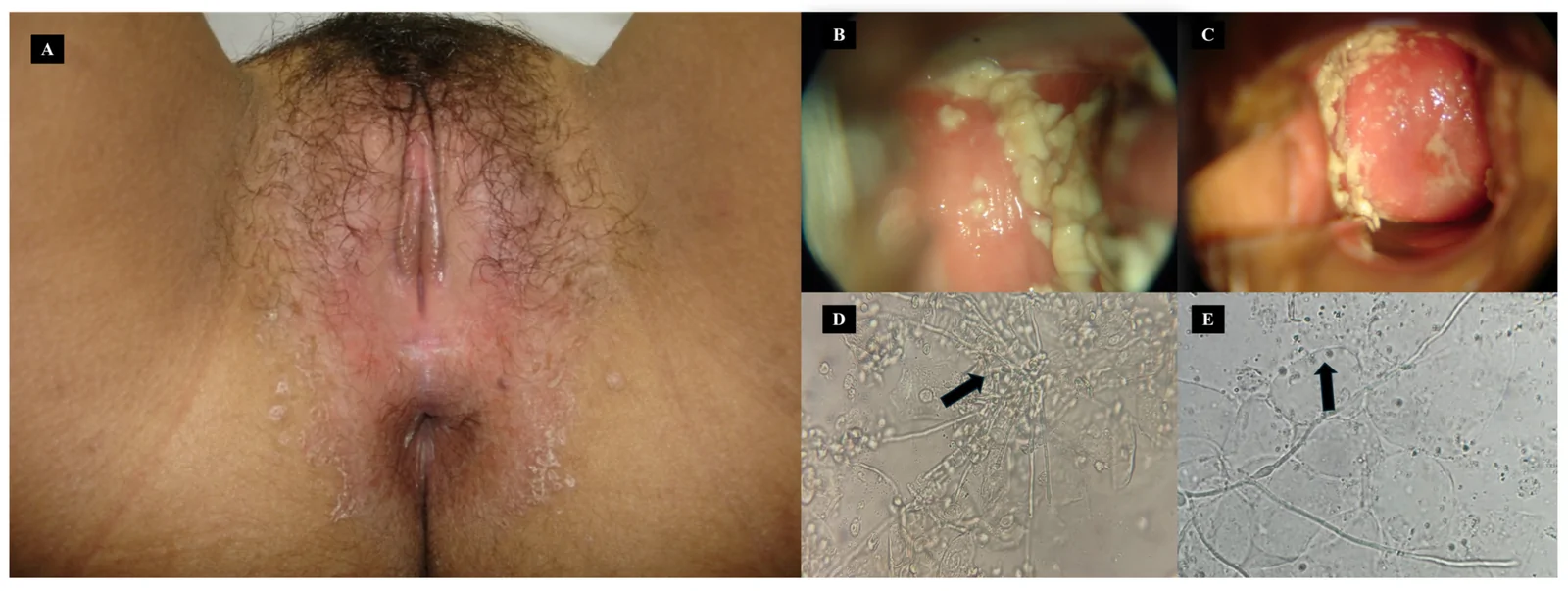
Representative clinical and laboratory findings in a pregnant woman presenting with vulvovaginal pruritus, burning, dysuria, dyspareunia, and vulvar edema. (**A**) Clinical examination demonstrating diffuse vulvar and perianal erythema with desquamation. (**B**,**C**) Speculum examination showing abundant thick, white, curd-like vaginal discharge adherent to the vaginal walls and cervix. The amine (whiff) test was negative, and vaginal pH was 4.5. (**D**) Fresh saline wet mount microscopy (×400) demonstrating fungal hyphae (arrow), consistent with vulvovaginal candidiasis. (**E**) Fresh saline wet mount microscopy (×400) showing numerous polymorphonuclear leukocytes (arrow) associated with abundant fungal hyphae, indicating a marked local inflammatory response. Clinical images were obtained from patients under the care of the authors and are presented with informed consent for publication.

Clinically, VVC is characterized by intense vulvovaginal pruritus, burning, dyspareunia, external dysuria, and a thick, white, curd-like vaginal discharge, frequently accompanied by vulvar erythema, edema, and fissures. Although these clinical manifestations are highly suggestive, laboratory confirmation is recommended whenever possible. Direct microscopic examination with potassium hydroxide (KOH) remains the primary diagnostic method, demonstrating budding yeast cells and/or pseudohyphae, whereas fungal culture is indicated for recurrent or refractory infections and when *Candida* species other than *C. albicans* species are suspected. The maintenance of a vaginal pH between 4.0 and 4.5 represents an important feature for distinguishing VVC from other causes of vaginitis [42,43,44,45]. The correlation between the characteristic clinical manifestations and the corresponding microscopic findings of vulvovaginal candidiasis is illustrated in Figure 7.

Although VVC is not as strongly associated with adverse obstetric outcomes as bacterial vaginosis, its high prevalence during pregnancy may substantially impair maternal quality of life. In addition, maternal genital colonization increases the likelihood of vertical transmission during vaginal delivery, contributing to neonatal oral candidiasis, diaper dermatitis, and other forms of mucocutaneous colonization. Recurrent episodes may also reflect persistent disturbances of the vaginal ecosystem or coexistence with other vaginal disorders, highlighting the importance of appropriate microbiological investigation and an individualized approach to management during prenatal care [42,43,44,45].

### 5.3. Trichomoniasis

Trichomoniasis is the most prevalent nonviral sexually transmitted infection worldwide and is caused by the flagellated protozoan *Trichomonas vaginalis*. The infection predominantly affects women of reproductive age and is particularly common among socially vulnerable populations and women living with human immunodeficiency virus (HIV). Beyond its sexual transmission, *T. vaginalis* exhibits a marked tropism for the vaginal epithelium, where it disrupts the vaginal microbiota, increases vaginal pH, and induces a local inflammatory response. Epidemiological studies have reported an association between *T. vaginalis* infection and an increased risk of HIV acquisition and transmission; however, these findings should not be interpreted as establishing a direct causal relationship [46,47,48,49].

**Figure 8:**
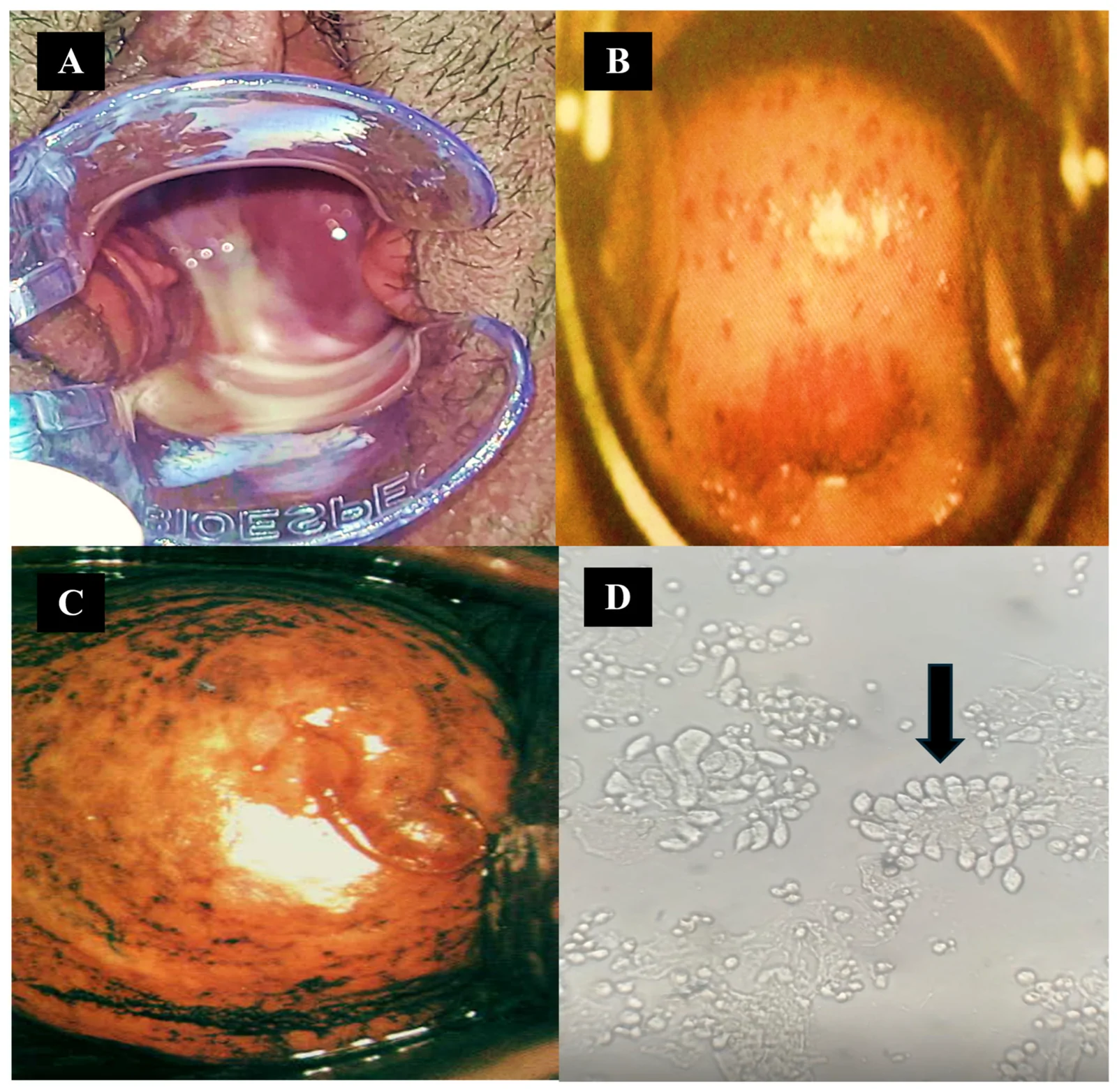
Clinical and microscopic findings in a 30-year-old pregnant woman presenting with yellow vaginal discharge and foul odor. (**A**) Speculum examination showing abundant yellow vaginal discharge. The amine (“whiff”) test was positive, and vaginal pH was 6.0. (**B**) Speculum examination demonstrating focal colpitis with the characteristic “strawberry cervix” appearance. (**C**) Schiller iodine test showing a “tiger-striped” pattern secondary to focal epithelial inflammation. (**D**) Wet mount microscopy (×400) demonstrating motile *Trichomonas vaginalis* (arrow) organisms associated with a marked inflammatory response characterized by numerous polymorphonuclear leukocytes. Clinical images were obtained from patients under the care of the authors and are presented with informed consent for publication.

Although most infected women remain asymptomatic, symptomatic trichomoniasis typically presents with abundant, frothy, yellow-green vaginal discharge; vulvovaginal pruritus; irritation; dyspareunia; and dysuria. The presence of punctate cervical hemorrhages, classically described as a “strawberry cervix,” is highly suggestive of the diagnosis but is observed in only a minority of cases. Nucleic acid amplification tests (NAATs) are currently considered the diagnostic method of choice because of their superior sensitivity and specificity, whereas wet mount microscopy, despite its rapid availability and low cost, has substantially lower diagnostic accuracy and should be interpreted within the appropriate clinical context [46,47,48,49]. Figure 8 illustrates the correlation between the characteristic clinical manifestations and the corresponding microscopic findings of trichomoniasis.

During pregnancy, trichomoniasis has been associated with an increased risk of adverse obstetric outcomes, including preterm premature rupture of membranes, preterm birth, low birth weight, delivery of small-for-gestational-age infants, and perinatal mortality. Although vertical transmission is relatively uncommon, neonatal infection may occur during vaginal delivery, resulting in colonization or infection of the respiratory or genitourinary tract. These complications are thought to arise primarily from parasite-induced inflammation and disruption of the cervical and vaginal mucosal barrier, reinforcing the importance of timely diagnosis and appropriate treatment of symptomatic pregnant women, particularly those at increased risk of obstetric complications [47,48,49,50].

### 5.4. Aerobic Vaginitis

Aerobic vaginitis (AV) is a distinct inflammatory vaginal disorder characterized by depletion or complete loss of *Lactobacillus* species and their replacement by aerobic and facultative bacteria, including *Streptococcus agalactiae*, *Escherichia coli*, *Enterococcus faecalis*, and *Staphylococcus aureus*. Although less prevalent than bacterial vaginosis, AV is of considerable obstetric importance because it elicits a pronounced inflammatory response within the vaginal epithelium. Unlike bacterial vaginosis, which is associated with relatively limited mucosal inflammation, AV is characterized by marked activation of the innate immune response, increased production of pro-inflammatory cytokines, and epithelial injury, all of which facilitate ascending infection and contribute to adverse pregnancy outcomes [51,52,53,54].

**Figure 9:**
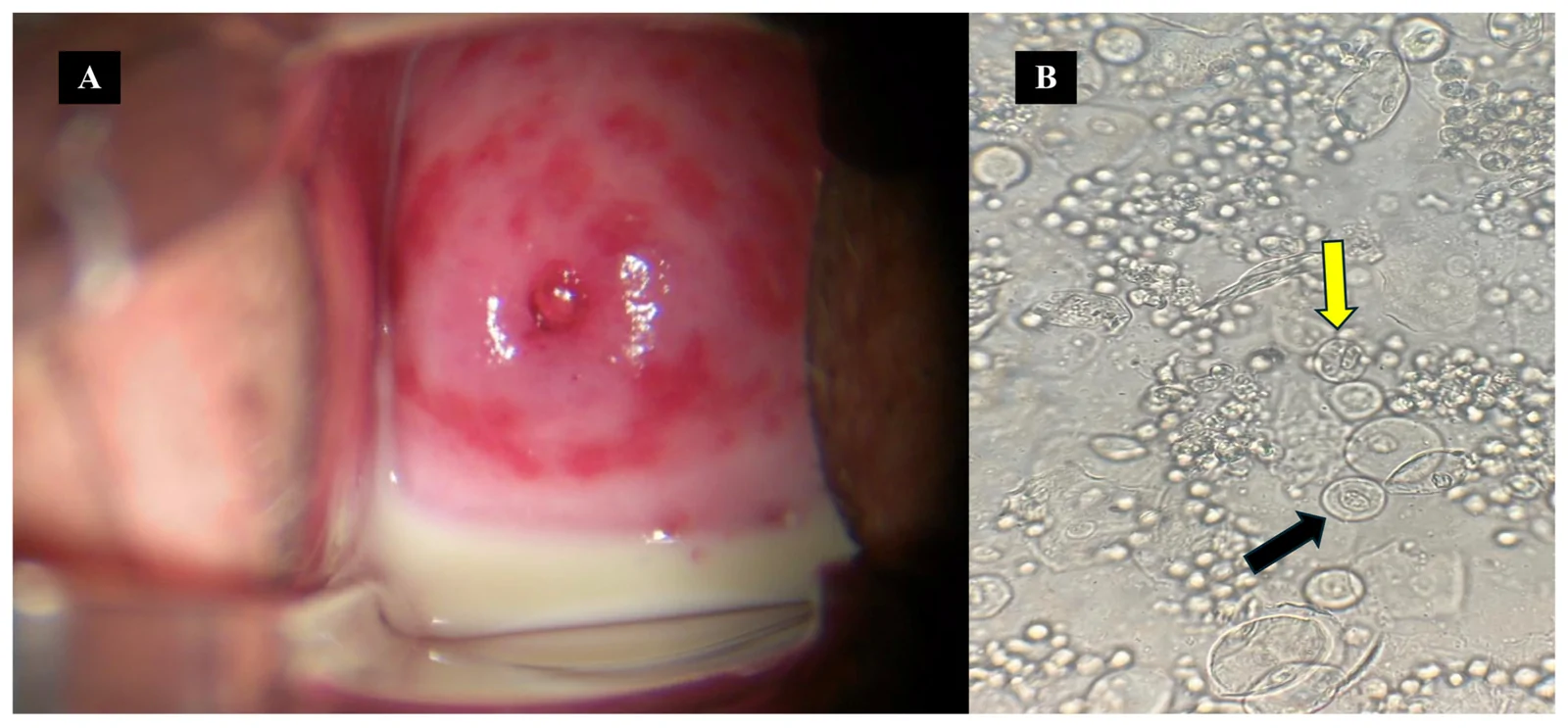
Clinical and microscopic findings of aerobic (desquamative) vaginitis during pregnancy. Representative clinical and laboratory findings in a pregnant woman presenting with vulvovaginal burning, yellow vaginal discharge, and dyspareunia. (**A**) Speculum examination demonstrating diffuse vaginal inflammation with erythema, petechial and ecchymotic lesions, and superficial erosions associated with yellow vaginal discharge. Vaginal pH was >4.5, and the amine (whiff) test was negative. (**B**) Fresh saline wet mount microscopy (×400) showing a marked inflammatory response characterized by numerous toxic polymorphonuclear leukocytes (yellow arrow), abundant parabasal epithelial cells (black arrow), and increased background microbiota, representing key microscopic parameters evaluated by the Donders scoring system for the diagnosis and grading of aerobic vaginitis. Clinical images were obtained from patients under the care of the authors and are presented with informed consent for publication.

Clinically, AV presents with thick yellow-green vaginal discharge, burning, dyspareunia, and vaginal discomfort, frequently accompanied by vulvovaginal erythema, edema, superficial erosions, and a vaginal pH typically exceeding 6.0. In contrast to bacterial vaginosis, the amine test is usually negative, providing a useful feature for differential diagnosis. Diagnosis relies primarily on microscopic examination of vaginal secretions using the Donders scoring system, which evaluates the lactobacillary grade, number of leukocytes, proportion of toxic leukocytes, presence of parabasal epithelial cells, and characteristics of the background microbiota. Each parameter is scored according to the degree of abnormality, and the individual scores are combined to generate a composite score reflecting AV severity, allowing classification as absent, mild, moderate, or severe aerobic vaginitis [51,52,53,54,55]. Although the Donders scoring system is widely used for the diagnosis and severity classification of AV, diagnostic criteria for AV remain less universally standardized than those established for BV, which may contribute to variability in prevalence estimates and comparisons across studies. The characteristic clinical and microscopic features of AV during pregnancy, including the findings incorporated into the Donders scoring system, are illustrated in Figure 9.

During pregnancy, AV has been associated with an increased risk of chorioamnionitis, preterm premature rupture of membranes, preterm birth, fetal infection, puerperal sepsis, and other maternal and neonatal infectious complications. These adverse outcomes are believed to result from the combined effects of high bacterial burden, an exaggerated local inflammatory response, and disruption of the vaginal epithelial barrier. Despite increasing recognition of its clinical relevance, the epidemiology of AV remains incompletely defined, and important gaps persist regarding the role of routine screening, optimal therapeutic strategies, and the impact of treatment on obstetric outcomes. Consequently, AV remains an important focus for future clinical and translational research [52,53,54,55,56].

## 6. Maternal, Fetal, and Neonatal Outcomes Associated with Vaginal Infections

The associations between vaginal infections during pregnancy and adverse maternal, fetal, and neonatal outcomes vary substantially according to the specific condition, reflecting differences in pathogenesis, inflammatory response, microbial behavior, and the strength of the available evidence. BV has the strongest and most consistent association with ascending infection and adverse obstetric outcomes, whereas trichomoniasis has also been associated with several adverse pregnancy outcomes. AV may contribute to obstetric complications through pronounced local inflammation and epithelial disruption, although the available evidence is more limited. In contrast, VVC is highly prevalent during pregnancy but has a substantially weaker and less consistent association with adverse obstetric outcomes. Accordingly, these conditions should not be considered equivalent in either their pathogenic mechanisms or their implications for maternal and perinatal risk [27,28,36,40,57,58]. The clinical impact of these infections is influenced by several factors, including the infecting microorganism, microbial burden, composition of the vaginal microbiota, maternal immune response, gestational age at infection, and the integrity of local defense mechanisms. Consequently, the same pathogen may result in markedly different clinical outcomes, ranging from asymptomatic colonization to severe maternal and perinatal complications. Among the adverse obstetric outcomes most consistently associated with vaginal infections are preterm birth, preterm premature rupture of membranes (PPROM), chorioamnionitis, intra-amniotic infection, spontaneous abortion, low birth weight, and delivery of small-for-gestational-age infants. BV demonstrates the strongest and most consistent association with these complications. In a meta-analysis of 18 prospective studies and control groups of clinical trials including 20,232 pregnant women, BV was associated with more than twofold higher odds of preterm delivery (OR 2.19, 95% CI 1.54–3.12) and increased odds of maternal infection (OR 2.53, 95% CI 1.26–5.08), with stronger associations with preterm delivery when BV was identified earlier in pregnancy [59]. Trichomoniasis has likewise been associated with adverse pregnancy outcomes. A systematic review and meta-analysis of 19 studies found increased odds of preterm delivery (OR 1.27, 95% CI 1.08–1.50), prelabor rupture of membranes (OR 1.87, 95% CI 1.53–2.29), and low birth weight (OR 2.12, 95% CI 1.15–3.91) among pregnant women with trichomoniasis [49]. In contrast, although VVC is highly prevalent during pregnancy, current evidence indicates a substantially weaker association with adverse obstetric outcomes. AV has also been associated with preterm birth, PPROM, chorioamnionitis, and neonatal infection, although robust pooled estimates remain unavailable [40,52,53,54].

Beyond obstetric complications, disruption of the maternal–fetal interface may directly affect fetal and neonatal health. Ascending infection and intrauterine inflammation expose the fetus to a pro-inflammatory intrauterine environment that has been associated with fetal inflammatory response syndrome, impaired fetal growth, low birth weight, and increased neonatal morbidity. During vaginal delivery, maternal genital infections may also facilitate vertical transmission of pathogenic microorganisms, contributing to neonatal respiratory or gastrointestinal colonization, oral candidiasis, neonatal sepsis, and other infectious complications that frequently require admission to neonatal intensive care units. Although the magnitude of these risks varies according to the underlying pathogen and the timing of infection during pregnancy, accumulating evidence underscores the pivotal role of the vaginal ecosystem in determining perinatal outcomes [40,49,57,58,59,60,61,62,63].

Maternal consequences may also extend into the puerperium. Although maternal sepsis secondary to lower genital tract infections is uncommon, vaginal pathogens may serve as the source of severe puerperal infections, particularly in women with PPROM, prolonged labor, operative vaginal delivery, cesarean section, or other conditions that facilitate ascending infection. Puerperal sepsis remains one of the leading causes of maternal mortality worldwide and is associated with septic shock, multiple organ dysfunction, prolonged hospitalization, intensive care admission, and increased fetal and neonatal mortality [61,62,63,64,65].

Collectively, these observations emphasize that preserving vaginal eubiosis is an important component of maternal and perinatal health. Early recognition of vaginal dysbiosis and genital infections, accurate microbiological diagnosis, individualized treatment, and implementation of preventive strategies have the potential to reduce adverse pregnancy outcomes. Nevertheless, important knowledge gaps remain regarding the identification of women who would benefit most from screening, the management of asymptomatic infections, and the development of microbiome-based interventions capable of preventing maternal, fetal, and neonatal complications [57,58,59,60,61,62,63,64,65].

## 7. Current Diagnostic Approaches for Vaginal Infections During Pregnancy and the Puerperium

Accurate diagnosis of vaginal infections during pregnancy and the puerperium requires an integrated approach that combines clinical assessment with laboratory confirmation, as symptoms and physical findings alone have limited diagnostic accuracy and considerable overlap among different etiologies. Clinical evaluation should include the characterization of vaginal discharge, odor, pruritus, burning, dyspareunia, associated urinary symptoms, sexual history, previous episodes, recent antimicrobial exposure, and gestational age. Speculum examination provides valuable information regarding the appearance of vaginal secretions, the degree of inflammation, cervical abnormalities, and the presence of vulvovaginal lesions. Measurement of vaginal pH remains a rapid, inexpensive bedside test that contributes to the differential diagnosis: values > 4.5 favor BV, trichomoniasis, and AV, whereas a pH ≤ 4.5 is more consistent with VVC [36,42,46,51,66,67,68].

Conventional laboratory methods continue to play a central role in routine clinical practice. Wet mount microscopy offers immediate identification of clue cells, motile *Trichomonas vaginalis*, budding yeast, pseudohyphae, leukocytes, and parabasal cells while simultaneously providing an assessment of the inflammatory response.

For BV, diagnosis is traditionally established using either the Amsel criteria or the Nugent score based on Gram-stained vaginal smears. The Amsel criteria require the presence of at least three of the following four findings: homogeneous thin vaginal discharge, vaginal pH > 4.5, a positive amine (“whiff”) test after addition of 10% potassium hydroxide, and the presence of clue cells on wet mount microscopy. Alternatively, the Nugent score, which remains the microbiological reference standard in both clinical practice and research, is based on the quantitative assessment of bacterial morphotypes on Gram stain, assigning a score from 0 to 10 according to the relative abundance of large Gram-positive rods (*Lactobacillus* spp.), small Gram-variable rods (*Gardnerella vaginalis* and *Bacteroides* spp.), and curved Gram-negative/Gram-variable rods (*Mobiluncus* spp.). Scores of 0–3 indicate normal vaginal microbiota, 4–6 represent an intermediate microbiota, and 7–10 are diagnostic of BV [66,67,68,69,70].

In contrast, AV is diagnosed using the Donders score, a standardized wet mount-based scoring system that grades disease severity according to the relative abundance of lactobacilli, the presence of aerobic bacterial flora, the number and toxicity of leukocytes, and the proportion of parabasal epithelial cells, thereby distinguishing AV from BV and other causes of vaginitis [66,67,68,69,70].

Culture retains an important role in selected clinical scenarios rather than as a first-line diagnostic method. It is particularly useful in women with recurrent VVC, suspected non-*albicans Candida* species, persistent symptoms despite appropriate therapy, or suspected antifungal resistance, allowing species identification and, when indicated, antifungal susceptibility testing. In contrast, culture has little diagnostic value for BV because organisms such as *Gardnerella vaginalis* and other anaerobes frequently colonize healthy women, making microbiological isolation alone insufficient to distinguish colonization from disease [66,67,68].

Over the past decade, molecular diagnostics have transformed the evaluation of vaginal infections. Nucleic acid amplification tests (NAATs) have become the preferred diagnostic method for *T. vaginalis* because of their substantially greater sensitivity compared with wet mount microscopy, while multiplex molecular panels now enable the simultaneous detection of BV-associated bacteria, *Candida* species, and *T. vaginalis* from a single vaginal specimen with excellent diagnostic performance. In addition to improving sensitivity and specificity, these assays facilitate the recognition of mixed infections, which are relatively common during pregnancy and may influence therapeutic decisions [66,67,71,72,73].

More recently, advanced molecular technologies have expanded the understanding of the vaginal ecosystem beyond conventional pathogen detection. Quantitative PCR permits estimation of microbial burden; matrix-assisted laser desorption/ionization time-of-flight mass spectrometry (MALDI-TOF MS) enables rapid species identification from cultured isolates; and next-generation sequencing (NGS), including 16S rRNA gene sequencing and shotgun metagenomics, provides comprehensive characterization of the vaginal microbiome. These approaches have revealed complex microbial communities, identified previously unrecognized microorganisms, and improved understanding of the relationships between community state types, dysbiosis, and adverse obstetric outcomes. Artificial intelligence-assisted bioinformatic analyses and machine learning algorithms are now being investigated to integrate microbiome profiles with clinical and laboratory variables, offering the potential for individualized prediction of dysbiosis, treatment response, and pregnancy complications [69,70,71,72,73,74].

Despite these advances, important barriers continue to limit the incorporation of these technologies into routine obstetric care, including cost, limited availability, lack of methodological standardization, and uncertainties regarding the clinical interpretation of microbiome profiles. Consequently, conventional clinical evaluation and validated laboratory methods remain the cornerstone of diagnosis. Emerging molecular technologies should therefore be viewed as complementary tools that enhance, rather than replace, clinical judgment, supporting more precise etiological diagnosis, antimicrobial stewardship, and individualized obstetric risk stratification in pregnant and postpartum women [69,70,71,72,73,74].

## 8. Evidence-Based Management of Vaginal Infections During Pregnancy and the Puerperium

The management of vaginal infections during pregnancy and the puerperium should balance microbiological cure with maternal–fetal safety while preserving the integrity of the vaginal ecosystem. Therapeutic decisions should consider the infecting microorganism, symptom severity, gestational age, risk of ascending infection, previous treatment history, and the physiological changes that characterize pregnancy and the postpartum period. Because untreated symptomatic infections may increase the risk of maternal morbidity and adverse obstetric outcomes, treatment is recommended for all symptomatic pregnant women. In contrast, routine screening and treatment of asymptomatic women remain controversial and are generally reserved for selected high-risk populations or specific clinical indications, reflecting the limited evidence that universal screening improves pregnancy outcomes [36,41,46,67,69,75,76,77,78].

The therapeutic recommendations presented in this section are based on current international guidelines, including those issued by the centers for Disease Control and Prevention, the World Health Organization, and other major professional societies, as applicable to each condition [36,41,46,67,69,75,76,77,78]. Current treatment recommendations are supported by substantial evidence regarding efficacy and safety during pregnancy. For BV, metronidazole is the preferred treatment because of its well-established efficacy and safety during pregnancy, whereas clindamycin constitutes an effective alternative when clinically indicated or when metronidazole is not tolerated. Metronidazole is also the treatment of choice for trichomoniasis and should not be withheld because of pregnancy, given the potential maternal and perinatal consequences of untreated infection. In contrast, VVC is preferably managed with topical azole antifungals administered for seven days, as prolonged local therapy provides higher cure rates than shorter regimens during pregnancy. Oral fluconazole, particularly at high doses or after repeated exposure during the first trimester, should generally be avoided because of reported associations with adverse fetal outcomes. Therapeutic management of AV remains less well standardized and should be individualized according to disease severity, with treatment directed toward the predominant aerobic pathogens while minimizing unnecessary disruption of the resident vaginal microbiota [36,41,46,67,69,75,76,77,78].

Antimicrobial resistance represents an increasing challenge in the management of vaginal infections. Although most *Trichomonas vaginalis* infections remain susceptible to nitroimidazoles, metronidazole-resistant isolates have been increasingly recognized and should be considered in women with persistent infection after appropriate treatment, once poor adherence and reinfection have been excluded. Such cases may require alternative or higher-dose nitroimidazole regimens and specialist-guided management. Antifungal resistance is also clinically relevant in vulvovaginal candidiasis, particularly in recurrent disease and infections caused by non-*albicans Candida* species, which may exhibit reduced susceptibility or intrinsic resistance to commonly used azoles. Persistent or recurrent symptoms should therefore prompt microbiological confirmation, species identification, and, when available, susceptibility testing to guide individualized therapy and avoid unnecessary or repeated empirical antimicrobial exposure [41,46,47,48,49,50].

The puerperium presents unique therapeutic challenges because the vaginal microbiome undergoes a prolonged transition toward recovery. Marked hypoestrogenism during lactation results in reduced glycogen availability, diminished *Lactobacillus* abundance, higher vaginal pH, and increased susceptibility to persistent dysbiosis. Consequently, restoration of vaginal eubiosis often requires several months and may extend throughout the breastfeeding period. Most antimicrobial regimens recommended for vaginal infections, including metronidazole and clindamycin, are compatible with breastfeeding, and fluconazole can be administered when clinically indicated. Therefore, postpartum management should focus not only on pathogen eradication but also on facilitating recovery of the vaginal ecosystem while minimizing unnecessary antimicrobial exposure [66,75,76,77,78,79].

Growing recognition of the importance of the vaginal microbiome has shifted the therapeutic paradigm from pathogen eradication alone toward restoration of microbial homeostasis. Adjunctive therapies containing selected *Lactobacillus* strains have shown encouraging results in reducing recurrence rates and accelerating re-establishment of a Lactobacillus-dominated microbiota, particularly following treatment for BV. However, the available evidence remains heterogeneous with respect to probiotic strains, formulations, treatment duration, routes of administration, study populations, and clinical endpoints. Consequently, current evidence does not support the universal recommendation of probiotics for the prevention or treatment of vaginal infections during pregnancy and the puerperium, and their use should be considered investigational or individualized pending more robust evidence from well-designed clinical trials. Other microbiome-directed strategies currently under investigation include vaginal microbiota transplantation, live biotherapeutic products, next-generation probiotics, bacteriocins and other microbial metabolites, phage-based therapies, and targeted modulation of host immune responses. Although these approaches remain largely investigational, they illustrate the ongoing transition toward precision medicine in the management of vaginal infections [76,79,80,81,82].

Future therapeutic strategies are expected to integrate molecular diagnostics, microbiome profiling, and individualized risk prediction to guide personalized interventions that optimize antimicrobial selection, reduce recurrence, preserve vaginal eubiosis, and ultimately improve maternal, fetal, and neonatal outcomes. As understanding of host–microbiome interactions continues to evolve, preservation of the vaginal ecosystem is likely to become a central therapeutic objective rather than merely a secondary consequence of antimicrobial treatment [76,79,80,81,82].

Prevention remains a fundamental component of the management of vaginal infections during pregnancy and the puerperium, extending beyond antimicrobial treatment to include strategies that preserve vaginal eubiosis and reduce the risk of recurrence. Current evidence does not support routine screening for bacterial vaginosis in asymptomatic pregnant women at low obstetric risk, as universal screening has not consistently demonstrated improvements in pregnancy outcomes. Instead, preventive efforts should focus on the prompt diagnosis and treatment of symptomatic infections, optimization of prenatal care, avoidance of unnecessary vaginal practices such as douching, judicious use of systemic antibiotics to minimize microbiome disruption, and patient education regarding genital hygiene and modifiable behavioral risk factors. Women with recurrent infections may also benefit from individualized follow-up and, when appropriate, adjunctive microbiome-directed therapies aimed at restoring a *Lactobacillus*-dominant vaginal environment. Collectively, these measures seek not only to prevent recurrent dysbiosis but also to reduce the risk of ascending infection, preterm birth, premature rupture of ovular membranes, postpartum endometritis, and other maternal and neonatal complications, reinforcing prevention as an integral component of comprehensive vaginal health care during pregnancy and the postpartum period [36,41,46,66,67,69,75,76,77,78,79,80,81,82].

**Figure 10:**
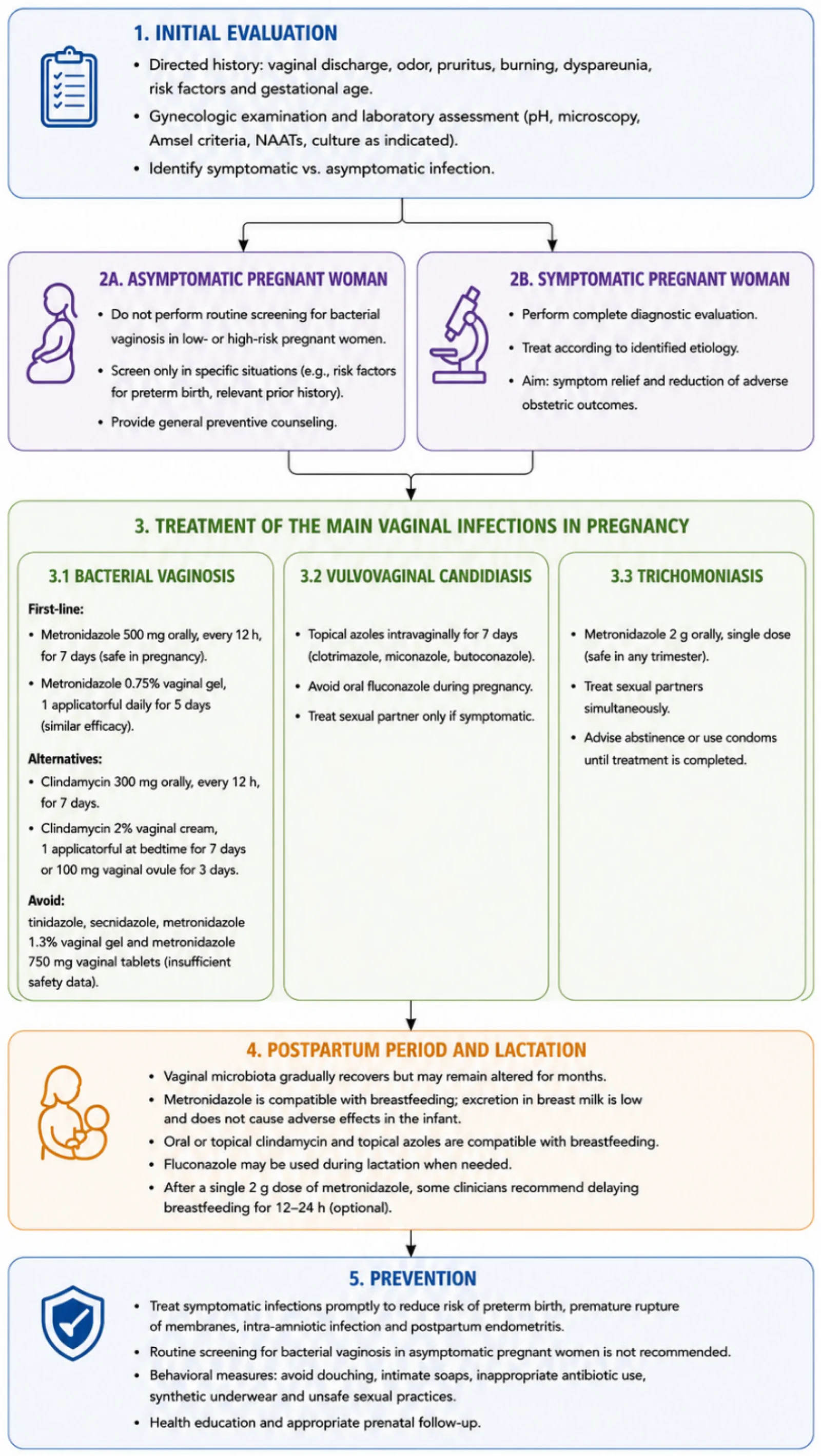
Evidence-Based Management of Vaginal Infections During Pregnancy and the Postpartum Period.

To facilitate the clinical application of the evidence discussed throughout this review, Figure 10 summarizes a practical, evidence-based approach to the diagnosis and management of the major vaginal infections during pregnancy and the puerperium. The algorithm begins with the initial clinical and laboratory evaluation, including symptom assessment, pelvic examination, vaginal pH measurement, microscopy, and etiological testing when indicated, and distinguishes the management of symptomatic and asymptomatic pregnant women, reflecting current recommendations against routine screening for asymptomatic bacterial vaginosis in most pregnant women. For symptomatic patients, treatment is guided by the identified etiology using pregnancy-compatible antimicrobial regimens, including metronidazole or clindamycin for bacterial vaginosis, topical azoles for vulvovaginal candidiasis, and metronidazole for trichomoniasis. The algorithm also highlights key considerations for the postpartum period and lactation, emphasizing the physiological restoration of the vaginal microbiota, medication safety during breastfeeding, and preventive strategies aimed at reducing recurrent dysbiosis and adverse obstetric outcomes. Rather than replacing individualized clinical judgment, this framework provides a concise overview of current evidence and guideline-based recommendations with the ultimate goal of eradicating infection, preserving vaginal homeostasis, and minimizing maternal, fetal, and neonatal complications [36,41,46,66,67,69,75,76,77,78,79,80].

While the overall therapeutic approach is well established, optimal management extends beyond antimicrobial selection alone. Treatment success depends on appropriate drug selection, patient adherence, recognition and management of adverse effects, prevention of reinfection, and timely identification of treatment failure or recurrent disease. Gastrointestinal adverse effects, including nausea, abdominal discomfort, and a metallic taste, are the most common reactions associated with metronidazole, whereas topical azoles are generally well tolerated, with only occasional transient local burning or irritation. These adverse effects are usually mild and rarely require treatment discontinuation. Persistent or recurrent symptoms should prompt reassessment of the original diagnosis, evaluation of treatment adherence, exclusion of reinfection, and consideration of alternative etiologies, antimicrobial resistance, or mixed infections. This approach is particularly important in women with recurrent or refractory vulvovaginal candidiasis, in whom fungal culture with species identification should be performed to identify non-*albicans Candida* species, which frequently exhibit reduced susceptibility to azole antifungals. During pregnancy, prolonged topical azole therapy (7–14 days) remains the preferred strategy for persistent disease, whereas oral fluconazole should generally be avoided. Cases refractory to standard treatment warrant specialist evaluation and individualized management based on the identified pathogen, antifungal susceptibility, and underlying predisposing conditions [36,41,46,67,69,75,76,77,78,79,80].

## 9. Research Priorities and Future Perspectives

Despite substantial advances in the understanding of the vaginal microbiome and its role in pregnancy, several fundamental questions remain unanswered. Most available evidence is derived from observational studies, limiting causal inference regarding the relationship between microbial alterations and adverse obstetric outcomes. Whether dysbiosis is a primary driver of pregnancy complications or a biomarker of an already perturbed maternal environment remains incompletely understood. Likewise, the biological mechanisms linking alterations in vaginal microbial communities to cervical remodeling, membrane weakening, placental inflammation, and spontaneous preterm birth require further mechanistic investigation.

Importantly, vaginal microbiome composition is influenced by population-specific and host-related factors. Differences in the prevalence of *Lactobacillus*-dominated community states and in the relative abundance of diverse anaerobic taxa have been reported across ethnic and geographic populations. These variations may reflect a complex interplay among host genetic background, environmental exposures, socioeconomic conditions, diet, sexual practices, hygiene behaviors, and access to healthcare, rather than ethnicity or geography as isolated biological determinants. Host genetic variation may also influence mucosal immunity, epithelial responses, and susceptibility to microbial colonization. Consequently, population context should be considered when interpreting vaginal microbiome profiles and their associations with reproductive and obstetric outcomes.

In this context, another important challenge is the remarkable interindividual variability of the vaginal microbiome. Factors such as ethnicity, geography, genetics, hormonal status, sexual behavior, nutrition, socioeconomic conditions, and environmental exposures contribute to substantial differences in microbial composition, making universal definitions of vaginal eubiosis and dysbiosis difficult to establish. Future studies should prioritize longitudinal, multi-ethnic cohorts integrating metagenomics, metatranscriptomics, metabolomics, proteomics, and host immune profiling to better characterize functional host–microbiome interactions throughout pregnancy and the puerperium.

Future randomized controlled trials of microbiome-directed interventions should address several important evidence gaps. Study populations should be sufficiently large and diverse to determine whether efficacy varies according to ethnicity, geographic origin, baseline vaginal community state, and other host or environmental characteristics. Standardized diagnostic criteria, microbiome sampling and specimen-processing protocols, sequencing methodologies, bioinformatic pipelines, and predefined clinical and microbiological endpoints are needed to improve reproducibility and facilitate meaningful comparisons across studies. The current methodological heterogeneity across these domains remains a major barrier to the reproducibility, comparability, and interpretation of microbiome findings and their translation into clinical practice. Trials should also incorporate longitudinal follow-up to determine the durability of microbiome restoration and recurrence after treatment, rather than relying exclusively on short-term microbial changes. Importantly, maternal, fetal, and neonatal safety should be systematically assessed, with follow-up extending into the postpartum period and lactation whenever appropriate. Future trials should therefore evaluate not only restoration of a Lactobacillus-dominated vaginal ecosystem, but also clinically meaningful outcomes such as symptomatic recurrence, ascending infection, preterm birth, premature rupture of membranes, postpartum infection, and neonatal morbidity. Such standardized and adequately powered trials will be essential to determine which microbiome-directed interventions can be safely translated into routine obstetric practice.

Significant therapeutic challenges also remain. Although antimicrobial therapy effectively eradicates many vaginal pathogens, recurrence rates remain high, particularly for bacterial vaginosis, highlighting the limitations of pathogen-directed treatment alone. Future interventions are expected to move beyond conventional antibiotics toward microbiome restoration strategies, including optimized probiotic formulations, live biotherapeutic products, vaginal microbiota transplantation, bacteriophage therapy, postbiotics, and targeted modulation of local immune responses. However, robust randomized clinical trials are still needed to define their efficacy, safety, ideal patient selection, and long-term effects during pregnancy and lactation.

Antimicrobial resistance should also be considered an important priority for future research. Further studies are needed to define the prevalence, geographic distribution, and molecular mechanisms of metronidazole resistance in *Trichomonas vaginalis* and azole resistance in *Candida* species, particularly non-*albicans Candida*. Prospective surveillance and standardized susceptibility testing are needed to determine the clinical relevance of emerging resistance patterns, identify predictors of treatment failure, and guide alternative therapeutic strategies. These efforts will be essential to support antimicrobial stewardship and preserve the effectiveness of available therapies.

The rapid expansion of molecular diagnostics and computational biology is also expected to transform clinical practice. Artificial intelligence combined with microbiome profiling may enable individualized risk prediction, early identification of women at increased risk of adverse pregnancy outcomes, and personalized therapeutic decision-making. Ultimately, future management strategies are likely to integrate clinical, microbiological, immunological, and multi-omics data into precision medicine approaches that preserve vaginal homeostasis while minimizing unnecessary antimicrobial exposure.

Finally, important implementation challenges remain, particularly in low- and middle-income countries, where limited access to molecular diagnostics, specialized microbiome testing, and novel therapeutics may hinder translation of emerging evidence into clinical practice. Bridging these disparities through affordable diagnostic tools, evidence-based prevention strategies, and globally applicable clinical guidelines will be essential to reduce the worldwide burden of vaginal infections and their maternal, fetal, and neonatal consequences.

## 10. Conclusions

The vaginal microbiome is a dynamic and specialized ecosystem that undergoes substantial physiological remodeling throughout pregnancy and the puerperium. During pregnancy, *Lactobacillus*-dominated communities contribute to vaginal homeostasis through maintenance of an acidic environment, epithelial barrier integrity, and modulation of local immune responses, whereas the postpartum period is characterized by reduced *Lactobacillus* abundance, increased microbial diversity, and gradual recovery influenced by hormonal changes and lactation. Disruption of this ecological balance may result in dysbiosis or vaginal infection and has been associated, with varying strengths of evidence, with adverse maternal, fetal, and neonatal outcomes.

BV, VVC, trichomoniasis, and AV represent clinically important vaginal conditions during pregnancy and the puerperium, but they differ substantially in their pathogenesis, inflammatory characteristics, diagnostic criteria, and associations with adverse obstetric outcomes. Current evidence-based clinical practice should therefore remain centered on accurate etiological diagnosis using validated clinical and laboratory methods, followed by condition-specific, guideline-based treatment. Such an approach is essential for symptom resolution, appropriate antimicrobial use, recognition of treatment failure or resistance, and minimization of unnecessary disruption of the resident vaginal microbiota.

An ecosystem-centered perspective complements, rather than replaces, this established pathogen-directed approach by recognizing the importance of microbial community structure, host–microbiome interactions, and restoration of vaginal homeostasis. This perspective is particularly relevant across the pregnancy–postpartum continuum, during which physiological changes in hormonal status and microbial composition may influence susceptibility to dysbiosis, recurrence, and recovery. However, preservation or restoration of the vaginal microbiome should not currently be interpreted as an indication for routine use of microbiome-directed interventions for which clinical evidence remains insufficient.

Emerging strategies—including next-generation probiotics, postbiotics, live biotherapeutic products, vaginal microbiota transplantation, microbiome biomarkers, multi-omics approaches, and artificial intelligence-assisted precision medicine—remain investigational. Although these approaches offer promising opportunities for improved diagnosis, risk stratification, prevention of recurrence, and individualized treatment, their efficacy, safety, durability, and clinical utility, particularly during pregnancy and lactation, require confirmation in well-designed and adequately powered clinical studies. Standardization of diagnostic criteria, microbiome sampling and analytical methods, and clinically meaningful outcomes will also be essential before these innovations can be translated into routine obstetric practice.

Thus, current management of vaginal infections during pregnancy and the puerperium should remain grounded in validated diagnostic methods, guideline-based therapy, and antimicrobial stewardship, while emerging microbiome-directed and precision-medicine approaches should be regarded as promising areas for future research rather than established components of clinical care. This distinction provides a clinically responsible framework for integrating advances in vaginal microbiome science while maintaining maternal, fetal, and neonatal safety as the central priority.

## Data Availability

The data presented in this study are available on request from the corresponding author.
